# Likelihood-Ratio-Test Methods for Drug Safety Signal Detection from Multiple Clinical Datasets

**DOI:** 10.1155/2019/1526290

**Published:** 2019-02-19

**Authors:** Lan Huang, Jyoti Zalkikar, Ram Tiwari

**Affiliations:** ^1^Mathematical Statistician in the Division of Biostatistics, Office of Clinical Evidence and Analysis, CDRH, FDA, Silver Spring, MD 20993, USA; ^2^Mathematical Statistician in the Division of Biometrics I, Office of Biostatistics, OTS, CDER, FDA, Silver Spring, MD 20993, USA; ^3^Division Director in the Division of Biostatistics, Office of Clinical Evidence and Analysis, CDRH, FDA, Silver Spring, MD 20993, USA

## Abstract

Pre- and postmarket drug safety evaluations usually include an integrated summary of results obtained using data from multiple studies related to a drug of interest. This paper proposes three approaches based on the likelihood ratio test (LRT), called the LRT methods, for drug safety signal detection from large observational databases with multiple studies, with focus on identifying signals of adverse events (AEs) from many AEs associated with a particular drug or inversely for signals of drugs associated with a particular AE. The methods discussed include simple pooled LRT method and its variations such as the weighted LRT that incorporates the total drug exposure information by study. The power and type-I error of the LRT methods are evaluated in a simulation study with varying heterogeneity across studies. For illustration purpose, these methods are applied to Proton Pump Inhibitors (PPIs) data with 6 studies for the effect of concomitant use of PPIs in treating patients with osteoporosis and to Lipiodol (a contrast agent) data with 13 studies for evaluating that drug's safety profiles.

## 1. Introduction

Meta-analysis approaches for multiple independent studies have become very popular in medical research. In many observational and/or clinical trial studies, meta-analysis can be performed using the study-level summary measures or patient-level information; for example, the studies can be integrated using a common statistical measure such as the study-level mean or effect size and computing a weighted average of this common measure using a statistical approach such as a fixed-effect model or a random-effects model [1]. The weights are usually related to the study-level sample sizes or within study variation but may depend on other factors. This type of approach is referred as the traditional meta-analysis and is being extensively used (as supportive) in the pre- and postapproval of drug products for evaluating their efficacy and safety. The traditional meta-analysis of many large and small clinical trials, published studies, registries, and large clinical and/or observational databases, for thorough evaluation of clinical efficacy endpoints such as the mean change in the weight-loss or blood-pressure and hazard ratio in survival comparison and clinical safety endpoints such as odds ratio, risk ratio, and absolute risk difference, has become a common practice for a modern-day pre- and postmarket clinical/observational studies [1, 2]. For example, a number of meta-analyses of rosiglitazone trials for patients with type-2 diabetes have been conducted to evaluate the risk for myocardial infarction (MI) and cardiovascular mortality [3], whereas in a meta-analysis of 15 clinical trials submitted to FDA during 1987–2012, Borges et al. [4] reviewed randomized withdrawal maintenance trials for major depressive disorder.

Using the traditional meta-analysis for safety evaluation, researchers can evaluate the point estimates and 95% confidence intervals for odds ratio or risk ratio of the drug-AE pair of interest from each study, and then combine the estimates through a fixed-effect model or a random-effects model, produce an overall estimate of the parameter of interest and its associated 95% confidence interval, and then display the results using a forest plot. Here, we intend to extend the exploration of using traditional meta-analysis to safety signal detection, where relative risks (RRs) are commonly used when the drug exposure information is available, and they are usually called the risk ratios. The relative event rates or proportional reporting rates are used when there is lack of drug exposure information, which is usually the case in passive surveillance of medical products. It is important to explore safety signals in each study; however, when studying safety signals, researchers usually collect information from many trials (or studies) since a single clinical study with focus on efficacy cannot provide enough information for safety events. The clinical studies, included in a large safety data or database, are usually independent studies with different protocols. It is possible that a signal detected in one study may not be detected in other studies due to variation across studies (in terms of sample sizes, study sites, personnel, patients enrolled, study time, and others).

Several methods have been developed for data mining or safety signal detection for exploring multiple drugs and AEs (for example, proportional reporting ratios [5], reporting odds ratios [6], likelihood ratio tests [7–9], and Bayesian methods [10–13]). However, these signal detection methods usually work on pooled large passive data and are not designed to incorporate the heterogeneity from multiple studies. Here, we propose new methods for drug safety signal detection (with an intent to control the type-I error and false discovery rate), for data with multiple studies, obtained from large observational databases such as FDA event reporting system (FAERS; https://open.fda.gov/data/faers/) or from clinical trial databases. The new methods utilize the regular likelihood ratio test (LRT) for signal detection [7] and consist of a two-step approach for exploring safety signals from multiple studies/sources. In the first step, the regular LRT is applied to the safety data by study and in the second step, the regular LRT test statistics from different studies are combined to derive an overall test statistic for conducting the global test at a prespecified level of significance, and if the global null is rejected in favor of the global alternative, the data provides evidence of a signal, overall.

The paper is organized as follows. In Section 2, we give a brief review of the basic LRT method for signal detection (regular LRT) and introduce several methods, based on regular LRT, for signal detection from multiple studies. In Section 3, the proposed LRT methods for signal detection are applied to two datasets for illustration: first one, to a dataset on concomitant use of PPI drugs for patients taking drugs treating osteoporosis, with interest in comparing two drug groups (PPI + placebo vs. placebo only) from 6 selected studies; and second one to a selected set of 13 published studies on Lipiodol (a contrast agent) with maximum dose of 15 mg. A simulation study is conducted to evaluate the performance of the LRT analysis methods for multiple studies in Section 4. We conclude Section 5 with a discussion.

## 2. Methods

### 2.1. A Summary of Regular LRT

The likelihood-ratio-test based method for signal detection developed originally for passive surveillance of large safety databases and is available to public for use in openFDA (https://open.fda.gov/tools/), called here as the regular LRT method, is a frequentist method based on multiple 2 × 2 tables [7]. For a particular AE *j*, of interest, there are *I*2 × 2 tables if there are a total of *I* drugs in the study. Here, the drugs are considered different rows, and the jth AE can be considered as a column (see Section 3.1). If, for a particular drug, one wants to compare many AEs, drug should be considered as a column variable and the AEs should be the rows (see Section 3.2).

Define *n*_*ij*_ as the cell count for ith row (e.g., drug) and jth column (e.g., AE) and assume that *n*_*ij*_∼^*ind*^Poisson(*n*_*i*._*p*_*ij*_),  *i*=1,…*I*, where *p*_*ij*_ is the reporting rate of ith drug for jth AE, and that ((*n*_.*j*_ − *n*_*ij*_)∼^*ind*^Poisson(*n*_.._ − *n*_*i*._)*q*_*ij*_),  *i*=1,…*I*, where *q*_*ij*_ is the reporting rate of all other drugs excluding ith drug for *j*th AE. Here, *n*_*i*._=∑_*j*=1_^*j*=*J*^*n*_*ij*_, *n*_.*j*_=∑_*i*=1_^*i*=*I*^*n*_*ij*_, and *n*_.._=∑_*j*_*n*_.*j*_. Dropping the suffix *j* in *p*_*ij*_ and *q*_*ij*_, assume that AE_*j*_ is fixed, the interest is to test the null hypothesis *H*_0_ : *p*_*i*_=*q*_*i*_=*p*_0_, against the alternative hypothesis that *H*_*a*_ : *p*_*i*_ > *q*_*i*_(*i*.*e.*, RR_*i*_=*p*_*i*_/*q*_*i*_ > 1) for at least one *i*, *i*=1, ⋯, *I*. The likelihood ratio statistic for Drug_*i*_ and AE_*j*_, as derived in [7], is

(1) $$\begin{array}{c} {\text{LR}}_{ij}=\frac{{\left(n_{ij}/n_{i.}\right)}^{n_{ij}}{\left(n_{.j}-n_{ij}/n_{..}-n_{i.}\right)}^{n_{.j}-n_{ij}}}{{\left(n_{.j}/n_{..}\right)}^{n_{.j}}}={\left(\frac{n_{ij}}{E_{ij}}\right)}^{n_{ij}}{\left(\frac{n_{.j}-n_{ij}}{n_{.j}-E_{ij}}\right)}^{n_{.j}-n_{ij}},\;i=1,\cdots ,I, \end{array}$$

where *n*_.._=∑_*i*=1_^*I*^*n*_*i*._ and *E*_*ij*_=*n*_*i*._*n*_.*j*_/*n*_.._.

The maximum likelihood ratio (MLR) test statistic, for the one-sided alternative, is

(2) $$\begin{array}{c} \text{MLR}={\text{max}}_{i}\left({\text{LR}}_{ij}I\left({\hat{p}}_{i}>{\hat{q}}_{i}\right)\right)={\text{max}}_{i}{\left(\frac{n_{ij}}{E_{ij}}\right)}^{n_{ij}}{\left(\frac{n_{.j}-n_{ij}}{n_{.j}-E_{ij}}\right)}^{n_{.j}-n_{ij}}I\left({\hat{p}}_{i}>{\hat{q}}_{i}\right), \end{array}$$

where the maximum is taken over *i*=1, ⋯, *I*. Since logarithm log(LR_*ij*_) is a monotonic (increasing) function of LR_*ij*_, so it is convenient to work with $\text{MLLR}={\text{max}}_{i}\left(\text{log}\left({\text{LR}}_{ij}\right)I\left({\hat{p}}_{i}>{\hat{q}}_{i}\right)\right)$.

The above formulation was constructed assuming there is no drug exposure information in the large postmarket safety database from passive surveillance system. In this case, “no drug exposure” usually refers to the fact that we may know how many adverse events are reported with respect to a certain drug in a passive surveillance system, but we may not know the number of patients who actually took the drug and the drug exposure information for each person. Therefore, *n*_*i*._ was used to serve as an approximation of total drug use and relative reporting rates were compared for such an analysis using data from FDA adverse event reporting system (FAERS; https://open.fda.gov/data/faers/).

When the drug exposure for ith drug (*P*_*i*_) is available, all *n*_*i*._ can be replaced by *P*_*i*_ and the relative risks can then be compared with available drug exposure information (see some definitions in Huang et al. [8]). Drug exposure information may be available in a legacy database including data from completed clinical trials or data from ongoing clinical trials (for safety monitoring purpose). In clinical trial data, the drug exposure for a patient is usually well-defined and prespecified as the total dose taken by the patient during the study, or the exposure time from a certain amount of drug. In some cases, we may not have well defined drug exposure information from completed clinical trials. For example, the precise drug exposure for the concomitant use of PPI is not collected in the studies included in Section 3.1, where we may have to impute the exposure with some reasonable assumptions.

Note that in order to detect signals using information from multiple studies, the drug exposure definition should be consistent and comparable across different studies considered in a single meta-analysis. More details will be discussed in the applications.

The log likelihood ratio statistic is then written as

(3) $$\begin{array}{c} \text{log}\left({\text{LR}}_{ij}\right)=\text{log}\left[\frac{{\left(n_{ij}/P_{i}\right)}^{n_{ij}}{\left(n_{.j}-n_{ij}/P_{.}-P_{i}\right)}^{n_{.j}-n_{ij}}}{{\left(n_{.j}/P_{.}\right)}^{n_{.j}}}\right]\\ =\text{log}\left[{\left(\frac{n_{ij}}{E_{ij}}\right)}^{n_{ij}}\right]+\text{log}\left[{\left(\frac{n_{.j}-n_{ij}}{n_{.j}-E_{ij}}\right)}^{n_{.j}-n_{ij}}\right]-\text{log}\left[{\left(\frac{n_{.j}}{P_{.}}\right)}^{n_{.j}}\right]\\ =n_{ij}\times \left(\text{log}\left(n_{ij}\right)-\text{log}\left(E_{ij}\right)\right)+\left(n_{.j}-n_{ij}\right)\times \left(\text{log}\left(n_{.j}-n_{ij}\right)-\text{log}\left(n_{.j}-E_{ij}\right)\right)-n_{.j}\times \left(\text{log}\left(n_{.j}\right)-\text{log}\left(P_{.}\right)\right),\;i=1,\cdots ,I, \end{array}$$

where *P*_._=∑_*i*=1_^*I*^*P*_*i*_, ∑_*i*=1_^*I*^*P*_*i*_/*P*_._=1, and *E*_*ij*_=*P*_*i*_*n*_.*j*_/*P*_._.

Since the distribution of MLLR test statistic under the null hypothesis is not tractable, a Monte Carlo procedure (MC) is used to obtain the empirical distribution of MLLR. The empirical distribution of MLLR under the null hypothesis can now be obtained by generating a large number of Monte Carlo samples for the cell-report counts (*n*_1*j*_, ⋯, *n*_*Ij*_) and for the *j*^th^ AE, using multinomial distribution (*n*_1*j*_, ⋯, *n*_*Ij*_) | *n*_.*j*_ ~ Mult(*n*_.*j*_, ((*n*_1._/*n*_.._), ⋯, (*n*_*I*._/*n*_.._))) with known *n*_.*j*_ as the total number of events. If the drug exposure is available, the distribution is then (*n*_1*j*_, ⋯, *n*_*Ij*_) | *n*_.*j*_ ~ Mult(*n*_.*j*_, ((*P*_1_/*P*_._), ⋯, (*P*_*I*_/*P*_._))). If the MLLR based on the observed data, MLLR_data_, is greater than the threshold value of MLLR_0.05_ (the upper 5^th^ percentile point of the empirical distribution), the null hypothesis is rejected with alpha = 0.05. The *p* value of MLLR can be calculated as

(4) $$\begin{array}{c} 1-\frac{{\text{rank of MLLR}}_{\text{data}}{\text{ among MLLR}}_{\text{data}}\text{ and MLLRs from the empirical distribution}}{1+\text{total number of simulation for the empirical distribution generation}}. \end{array}$$

The drug associated with MLLR_data_ is then the most significant signal detected.

### 2.2. LRT Analysis Approaches for Signal Detection from Multiple Studies

Here, we propose several LRT approaches based on the regular likelihood ratio test (LRT) for safety signal detection with multiple studies. Note that in the following, logLR_*ijs*_ or logLR_*is*_ can be calculated by the formula described in Section 2.1 by study.

#### 2.2.1. Analysis of Pooled Data from Several Studies Using Regular LRT

Suppose there are a total of S studies or datasets. Let *n*_*ij*_(=∑_*s*_*n*_*ijs*_) denotes the total of event/report counts for ith drug and jth AE, summed over all the S studies (note that the subscript *i* is used for drug here and that one can define the row as drug or AE depending on the interest). Using this definition of “pooled” *n*_*ij*_, we can apply the regular LRT to detect the drug signals. However, the regular LRT applied to the pooled data may not control the type-I error as the Monte Carlo simulation for obtaining the empirical distribution of the test statistic is carried out based on the pooled data, but not the study-level data. We observed this issue in the simulation study.

Another issue with this analysis of pooled studies is that it does not address the study to study variation, that is, heterogeneity of studies. Study heterogeneity may come from different sources including study designs (prospective versus retrospective), different endpoints, different distributions of effect modifiers, and different source of data. Therefore, the analysis of the pooled studies without considering the heterogeneity may lead to biased results. This method is also vulnerable to Simpson's paradox [14, 15] and should be used with caution. For example, in a medical study for evaluating kidney stone treatment [16, 17], the paradoxical conclusion is that treatment A is more effective when used on patients with small stones and also when used on patients with large stones, yet treatment B is more effective on all patients (combined data).

In the following subsections, two LRT approaches for incorporating study-level heterogeneity are presented.

#### 2.2.2. Maximum of MLLR Statistics from Multiple Studies (MMLR)

Assume there are a total of S studies (with similar patients and objectives and are relevant for the purpose of current active/passive surveillance safety study), we define MLLR statistic for a fixed AE (*j*) of interest and sth study is MLLR_*s*_=max_*i*_(log(LR_*ijs*_))=max_*i*_(log(LR_*is*_)) dropping the suffix *j*. Then, the test statistic for testing the global null hypothesis versus the global alternative hypothesis is the maximum of MLLR_*s*_ over all studies defined by MMLLR=max_*s*_(MLLR_*s*_). The empirical distribution of MMLLR can be obtained by Monte Carlo simulation by generating the null data with *n*_.*s*_ and *P*_*is*_ from observed data and with the same relative risk for all rows from each study, *s*=1,…, *S*, and then calculating MMLLR=max_*s*_(max_*i*_(log(LR_*is*_)). Like the regular LRT, MMLR controls the type-I error.

A drug with MMLLR from observed data (for a particular study) is a signal if the related *p* value (the rank of the MMLLR from the observed data among the MMLLR values obtained from empirical data divided by the total number of empirical data) is less than a prespecified significance level (such as 0.05). Furthermore, if interested, we can identify secondary drug-study combinations as signals with logLR values (log(LR_*is*_),  *i*=1, ⋯, *I*, *s*=1, ⋯, *S*) as the second largest, third largest, and forth largest values among all values for the drug-study combinations.

#### 2.2.3. Weighted LRT Using Total Drug Exposure as Weight (wLRn)

In this subsection, we assume fixed jth column and drop the suffix *j* in the following derivations.

Let *P*_*is*_ be the total drug exposure for ith drug in sth study. Then, the weighted LRT statistic, based on the total drug exposure, is defined as wLR_*i*_=(∑_*s*=1_^*S*_*i*_^*P*_*is*_ log(LR_*is*_))/(∑_*s*=1_^*S*_*i*_^*P*_*is*_), where *S*_*i*_ denotes the number of studies for the ith drug, and note that *S*_*i*_, *i*=1, ⋯, *I* could be different for different rows. wLR_*i*_ can be interpreted as the weighted average of logLR from different studies for ith row with weight *P*_*is*_.

The test statistic for testing the global null hypothesis versus global alternative hypothesis is then defined as MwLR=max_*i*_(wLR_*i*_), where the maximum is obtained over all drugs, *i*=1, ⋯, *I*.

For statistical inference of wLRn method, the simulated null datasets are generated from a multinomial distribution with *n*_.*s*_ and *P*_*is*_ from observed data and with the same relative risk for all rows by study. The empirical distribution of wLR is formed by the 10,000 wLR_sim_ obtained from 10,000 simulated null data. The *p* value of the wLR_obs_ is obtained by comparing the wLR_obs_ with the 10,000 wLR_sim_ values from the Monte Carlo process:

(5) $$\begin{array}{c} p\text{ value}=\frac{\#{\text{ of times wLR}}_{\text{sim}}>{\text{wLR}}_{\text{obs}}}{10,000}. \end{array}$$

If the wLR_obs_ for ith drug (row) has *p* value <0.05, then the ith drug is a signal. After detecting the global signal, we can move to the 2nd largest, 3rd largest logLR or weighted logLR values, and so on for secondary signals.

In summary, the statistics discussed in Section 2.2 are presented in Table 1.

## 3. Applications

We illustrate the use of the LRT methods by applying them to two datasets with multiple studies for safety signal exploration. The first data is hypothetical, but based on real situation in the PPI data from FDA legacy database. The second data includes 13 published clinical studies on Lipiodol (a contrast agent) from literature search. In both examples, we tried to include studies with similar features for fair comparison (such as similar patients, similar drugs, and similar objectives).

### 3.1. Analysis of PPI Data with Two Drugs and a Composite AE

Proton Pump Inhibitors (PPIs) are a class of drugs that decrease gastric acid secretion through inhibition of the proton pump. It has been found that PPIs are associated with increased risk of hip fractures (adverse event) [18, 19]. Huang et al. [8] evaluated if the concomitant use of PPIs reduced the efficacy of test drugs intending to treat osteoporosis among targeted patients, using clinical trial data from FDA/OTS/OCS legacy database. That database contained data from 10 trials (including single-arm studies, two-arm studies, and three-arm studies). One medication (test drug for treating osteoporosis, active control, or placebo) will be given to patients in one arm, and PPIs were given to patients in different arms concomitantly. The sample sizes of the trials range from hundreds to more than thousands. The main focus was on the composite AE (AEOST as defined in Huang et al. [8], Appendix A1), which includes many AE terms related with osteoporosis symptoms. After further examination of this data, we noticed that one trial does not have placebo arm, one trial has placebo arm but does not have subjects with concomitant PPIs, and two trials do not have AEOST event reported in placebo + PPIs (PLandPPI) or placebo only (PL) groups. For illustration, we selected 6 trials with AEOST events reported and with partial subjects taking concomitant PPIs in the placebo arm. Note that the patients were randomized into test drug, active control, and placebo arms in those trials. The effect of PPIs and the other drugs (test drug and active control drugs) cannot be separated if they were used together in test drug arm and active drug arm. In the following, we illustrate the analysis of safety signals using the hypothetical data with 6 studies, which reflects the data pattern of the PPI clinical data for comparing PLandPPI and PL.

Two AEs considered here are the 1st occurrence of AEOST (denoted by 1occ) and repeated occurrences of AEOST (denoted by allocc). We evaluate the relative risks of the 1st occurrence of AEOST (or repeated occurrences of AEOST) for patients in PLandPPI group with exposure of placebo and concomitant PPIs vs. patients in PL group with exposure of placebo only. For 1occ analysis, *n*_*is*_ is the number of events for ith row (drug: placebo and PPIs together or placebo only) and sth study when one subject having only one event (1st occurrence of the repeated AEOST); *P*_*is*_ is the exposure (sum of the exposure times in units of person-day) to the 1st occurrence of AEOST from all subjects) for ith drug (row) and sth study. For allocc analysis, *n*_*is*_ is the number of events for ith drug (row) and sth study when one subject has several repeated events for one AE such as AEOST, and *P*_*is*_ is the exposure (sum of the drug exposure time from all subjects) for ith drug (row) and sth study. Note that a subject's exposure time here is defined to be the time period of the subjects with placebo in PL group (time from taking placebo to end of the study or drop-off). The exposure of concomitant PPIs in PLandPPI group is not well recorded and is always shorter than placebo period; therefore, we assume that the exposure of placebo and concomitant PPIs for subjects in PLandPPI group is simply the period of placebo exposure. The actual dose and exposure time of concomitant PPIs may vary by patient and the pattern may not be consistent with the total placebo exposure in the PLandPPI patients, which may introduce bias in evaluating the relative risk of PPIs together with placebo vs. placebo only.

**Figure 1:**
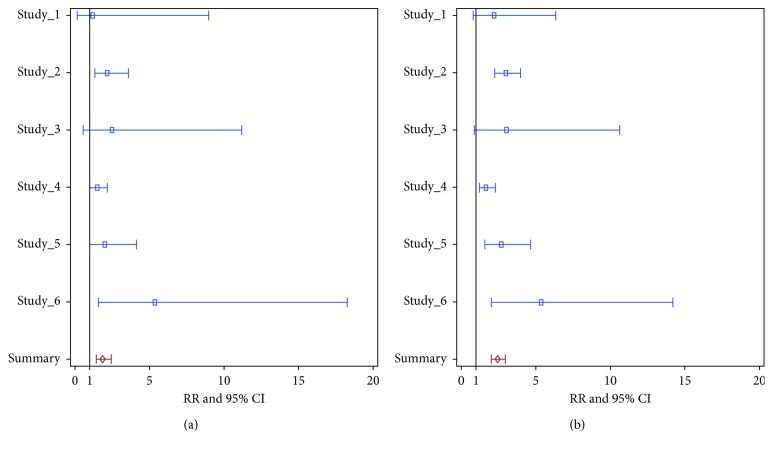
Forest plot of relative risk and 95% CI by study and summary (integrated using fixed effect model) relative risk using traditional meta-analysis methods (1occ analysis (a) and allocc analysis (b)).

Using traditional meta-analysis based on relative risks of safety issues, one may obtain an overall relative risk and 95% CI using fixed-effect or random-effects models (Borenstain et al. [1], chapters 11 and 12). The *τ*^2^ is 0 for the 1occ analysis and 0.07 for the allocc analysis. Therefore, the integrated results from fixed-effect model and random-effects model are almost the same. The overall relative risk and 95% CI is 1.87 (1.43, 2.45) for the 1occ analysis and 2.44 (2.02, 2.94) for the allocc analysis. The results are shown in Figure 1 by a forest plot.

We also analyzed these data using the LRT methods, namely, simple pooled analysis with regular LRT, MMLR, and wLRn. Note that the application with two drugs can be easily extended to multiple drugs using the step-down procedure in LRT analysis methods (not traditional meta-analysis methods). For example, if one drug (Drug A) vs. other drugs is a signal, another drug (Drug B) could be a secondary signal if the value of the test statistic has a *p* value smaller than 0.05 when there are more than two drugs.

For simple summary, the events and the relative risks (rr) of PLandPPI and PL with 95% confidence intervals by study are shown in Table 2. The results from regular LRT on individual study and the LRT analysis methods for multiple studies together are shown in Table 3. The 95% threshold in Table 3 is the 95% percentile of the empirical distribution of the related STAT.

The individual study analysis shows that the findings of the signals may vary in different studies with various levels of signal strength. The simple pooled analysis without considering the study variation and the MMLR and wLRn methods each considering the study-level variability have consistent results (AEOST is a signal for PLandPPI group when compared with PL only group). MMLR provides the strongest global signal of AEOST (along with the related study) as the integrated result. Stronger signal patterns were observed for the repeated occurrences analyses due to the large sample sizes. AEOST tends to be a signal for subjects taking concomitant use of PPIs (in PLandPPI group).

From the MMLR method, the most significant global signal of AEOST (1st occurrence or repeated occurrences) in subjects taking concomitant PPIs (PLandPPI group) comes from the 2nd study (*s* = 2). This signal for repeated occurrences is also seen in 4th study (*s* = 4, with *p* value 0.006), 5th study (*s* = 5, with *p* value 0.006), and 6th study (*s* = 6, with *p* value 0.009). The observed logLR for studies 2, 4, 5, and 6 are all greater than the threshold of 2.47 for the analysis of repeated occurrences (allocc).

### 3.2. Analysis of Lipiodol Data with One Drug and Multiple AEs

Lipiodol (labeled Ethiodol in the USA), also known as ethiodized oil, is a poppyseed oil used by injection as a radiopaque contrast agent that is used to outline structures in radiological investigations [20, 21]. It is used in chemoembolization applications as a contrast agent in follow-up imaging [22].

In order to detect possible safety signals to document the safety of Lipiodol when it is used for selective intraarterial use for imaging liver lesions in adults with known hepatocellular carcinoma (HCC), thirteen studies (articles) were identified with a maximum dose of Lipiodol as 15 ml recommended in the drug label, from more than 100 articles included in NDA 09190/S-024 submission (https://www.accessdata.fda.gov/scripts/cder/drugsatfda/). The actual doses for different subjects varied. However, the maximum dose was reported to be 15 ml for all subjects in those selected studies. The subjects in the 13 studies are all adults (average age ranging from 45 to 69). The year which the 13 articles published ranges from 1993 to 2009. The number of subjects in the studies ranges from 11 to 257. There are a total of 27 AEs reported in all the 13 studies for the drug considered (Lipiodol).

The number of subjects with a particular AE (*n*_*is*_) is reported by study, note that one subject may have multiple AEs reported. Since the exact drug exposure time is unknown for each subject from the articles, we assumed that the drug exposure is the same for each subject and that it is one unit. The total drug exposure by study (*P*_*is*_) is then the total number of subjects in each study, which is the same for all rows in this case (*P*_*is*_=*P*_*s*_).

Applying the regular LRT to the individual study and the LRT methods to all the 13 studies (with a total of 27 AE terms), the detected signals are shown in Tables 4 and 5. When the observed STAT (obsstat) is greater than the 95% threshold obtained from the empirical distribution under the null hypothesis of no signals, the related AE is a signal detected.

When interpreting the detected signals, one can consider lumping together similar AEs (with different AECODE codes) to form a group. For example, postembolization syndrome (PES) (with the definition from http://radiopaedia.org/articles/post-embolisation-syndrome-1), including AECODE codes FEVER, VOMITTING, NAUSEA, and ABDOMINAL PAIN, is detected by all LRT analysis methods (Table 5).

The detected signals varied in the individual study analyses (Table 4). By the simple pooled method and wLRn method, three AEs (all in PES group) are detected as signals with *p* value less than 0.05 (Table 5). All the signals are integrated signals by considering the information from all the 13 studies.

By the MMLR method, 21 AE-study combinations are detected as signals. PES is the most significant global signal among all the signals. There are 11 AEs among the signals (4 in PES group) ignoring the studies.

### 3.3. Summary of the Two Examples

We presented the two examples here for showing the performance of the proposed methods on two different types of datasets. Both examples consider data from clinical trials with some exposure information. The AE signals detected can be called signals with higher relative risk. Same kind of applications can be conducted for data from passive surveillance system by evaluating reporting rates (see reference [7]), because there is no exposure information and one cannot evaluate relative risk. Without exposure information, the formula used in computing the likelihood ratios (Section 2.1) will be different with different denominators.

The example of PPIs includes patients who were treated with multiple drugs and with exposure over time (patients may receive different doses at different visits). The AE studied (AEOST) in this example is an AE with many terms associated with osteoporosis. We may observe many repeated reports of the AEOST during the exposure duration. There are only two drug groups (drug groups as rows with *i*=1,2) and one selected composite AE (AEOST) for comparison. This is a simple case in signal detection and very similar to the set-up for traditional ways of data analysis in clinical trials. In this example, we compared the two drug groups with the fixed AE (AEOST).

In contrast, the example of Lipiodol is different. Contrast agents are used in discrete bursts, and many patients have only a single exposure to the drug. Therefore, if the dose of the one-time injection is similar for each patient, we can assume that the drug exposure is the same for each subject and that it is one unit. Then, *P*_*i*_ can be imputed with number of patients without more information on the exposure. In this example, we have 27 AEs (AEs as rows, *i*=1, ⋯, 27) and one drug of interest. The purpose of signal detection is to identify the AEs with high relative risks by comparing one AE vs. other AEs for Lipiodol. There are a total of 27 comparisons (not 2 comparisons such as the one in the first example). The proposed LRT method can handle the multiple comparisons here with false discovery rate (FDR) controlled [7]. Traditional meta-analysis evaluating risk ratios can be applied to the PPI data with two drug groups for comparison (two rows), but may not be applied to the Lipiodol data with more than two rows due to the inflated type-I error and FDR, in the presence of multiple comparisons.

## 4. Simulation

A simulation study is conducted with focus to evaluate the performance of the LRT analysis methods, discussed in Section 2, for data with multiple studies and drug exposure information available. The performance of tradition meta-analysis on risk ratio is also explored in simulated data with two rows.

### 4.1. Simulation Assumptions and Parameters

We simulate data using the information on the total number of studies, total number of rows, *n*_.*s*_, and *P*_*is*_ from the datasets used in the illustration (see the cases in Table 6), with equal relative risks for the data generation under the global null hypothesis (without any safety signals by study) and with different relative risks associated with different rows (for example, assigning the higher relative risk to the 1st row for data generation under the global alternative hypothesis). Note that each row corresponds to a drug or an AE. For example, row corresponds to a drug in Illustrations 3.1 and an AE in Illustration 3.2.

If the relative risks are the same for different rows, for each study, the simulated null data are generated from the following multinomial distribution (dropping suffix *j*):

(6) $$\begin{array}{c} \left(n_{1s},\cdots ,n_{Is}\right)\;\left|\;n_{.s}\right.∼\text{Mult}\left(n_{.s},\left(\frac{P_{1s}}{{\sum^{}}_{i=1}^{I}\left(P_{is}\right)},\cdots ,\frac{P_{Is}}{{\sum^{}}_{i=1}^{I}\left(P_{is}\right)}\right)\right). \end{array}$$

If the first row is a signal, with a higher relative risk, for each study, the simulated data (under global alternative) are generated from the following multinomial distribution:

(7) $$\begin{array}{c} \left(n_{1s},\cdots ,n_{Is}\right)\;\left|\;n_{.s}\right.∼\text{Mult}\left(n_{.s},\left(\eta_{1s}\frac{P_{1s}}{{\sum^{}}_{i=1}^{I}\left(\eta_{is}P_{is}\right)},\cdots ,\eta_{Is}\frac{P_{Is}}{{\sum^{}}_{i=1}^{I}\left(\eta_{is}P_{is}\right)}\right)\right), \end{array}$$

where *η*_1*s*_(*P*_1*s*_)/(∑_*i*_^*I*^(*η*_*is*_*P*_*is*_))+⋯+*η*_*Is*_(*P*_*Is*_)/(∑_*i*_(*η*_*is*_*P*_*is*_))=1. The relative risk of the first row vs. all other rows for sth study with *η*_*is*_=1, *i*=2, ⋯, *I*, is simply *η*_1*s*_. The values of *η*_1*s*_ (same for different studies) in this simulation are selected to be 1, 1.2, 1.5, 2, and 3 (results with *η*_1*s*_=1.2 will not be shown in Table 6, but the powers are low for the scenarios with *η*_1*s*_=1.2). *η* values may vary by study too (for example, *η*_1*s*_=1.5, 1.2, 3,1,1.3, 2 for studies 1 to 6, respectively, in the scenario with rr21 in Table 6).

The results for type-I error and power calculations, for different scenarios with equal relative risks (under global null) and different relative risks (under global alternative), are presented in Table 6. The drug exposure information *P*_*i*_ by row is obtained from the real data discussed in Section 4 (such as sim01occ, sim0allocc, and sim0lip). The drug exposure and case information are the same for scenarios sim01occ (null data) and sima1occ (alternative data), with only difference in the relative risks. The same rule was applied to all other null data and alternative data generation.

The total number of replications is 10,000 for the scenarios for type-I error evaluation and 1000 for the scenarios for power evaluation, respectively. The power is defined as the number of times the null hypothesis (that there is no signal detected in each study) is rejected, divided by the total number of replications. When the data are generated with the assumption of no signals, the power becomes type-I error.

### 4.2. Simulation Results

As shown in Table 6, the type-I error (or FDR) for data without any signals in each study stays low for wLRn and MMLR. The type-I error for the pooled method is slightly higher than the other methods with values up to 0.07 (not controlled). This is because the null data (counts) are generated from multinomial distribution by study and then they are simply added over all studies. The pooled method is then applied to the pooled data (observed), and the empirical distribution of statistics for decision making is obtained by Monte Carlo procedure based on the pooled data, but not on the study-level data. The other two LRT analysis methods controls the type-I error since both their statistics and the empirical distributions are based on study-level data and then are combined using different weighting approaches.

The power in Table 6 is highest for pooled method, and moderate for wLRn and MMLR methods. The MMLR method is more conservative than wLRn. The power values increase with the increase of relative risk values assigned to the 1st row. Usually, the power reaches 0.7-0.8 when the relative risk becomes 2 or 3, for all methods. The sample size in scenario simaallocc is larger than the scenario sima1occ; therefore, the power values are higher for scenarios simaallocc with different relative risks. In the scenario with rr21 (with different *η* values by study), the pooled analysis no longer has the largest power.

Traditional meta-analysis (Borenstein et al. [1]) with test based on normal assumption (Z statistic) and the null hypothesis that the mean effect is 1 (relative risk case) or 0 (log of relative risk) is applied to several simulated data scenarios with two rows for power and type-I error evaluation. If the *p* value (from the standard normal *Z* test) is less than 0.05, we reject the null hypothesis of relative risk (PLandPPI vs. PL) as 1. The type-I error is 0.067 and 0.070 for scenarios sim0allocc and sim01occ, respectively. The results reflect the inflated error of the traditional meta-analysis for data with two rows (one comparison). With more than two rows (multiple comparison) in the data, we expect bigger type-I error from the traditional meta-analysis.

The power values of the traditional meta-analysis are 73%, 98%, and 100% for scenarios simaallocc with true relative risks for the 1st row as 1.5, 2, and 3, respectively. The powers for sima1occ scenarios are smaller than the simaallocc cases due to smaller sample size, but reasonably large for cases with relative risk 2 or above. When the data were generated with varying relative risks for different studies (scenarios simaallocc and sima1occ with rr21), the powers from the traditional meta-analysis are very low (about 5%).

## 5. Discussion

In summary, the analysis using regular LRT on pooled data, MMLR, and weighted LRT method (wLRn) identifies signals for ith row (a drug or an AE) by incorporating the information from different studies. In addition, with MMLR method, one can identify the global signal(s) for ith row (a drug or an AE) along with the studies containing that global signal(s). Multiple signals can be detected with step-down process imbedded in the LRT method for wLRn and MMLR methods.

The traditional meta-analysis methods obtain a summary statistic based on the study-level statistics such as relative risk (can also called risk ratio) by fixed-effect or random-effects models or other weighting methods. There are two steps in the traditional methods: first, obtaining the study-level statistic (odds ratio or risk ratio), and second, obtaining the summary statistic for overall evaluation using the study-level statistic. One may then use a normal approximation for the confidence interval construction and testing for statistical significance using the summary statistic. The two-step approach is also used by the proposed LRT methods in a different way of exploring safety issues. However, in LRT methods (MMLR and wLRn), the study-level statistics are logLR. Monte Carlo (MC) simulation is used for testing for significance of the summary statistic. The use of logLR and a step-down process for identifying secondary signals with smaller logLR values and the nonparametric MC simulation for empirical distribution of the logLR or summary of logLR using null datasets together controls type-I error and FDR. In practice, one may consider conducting the traditional meta-analysis and the proposed signal detection method together in safety evaluation from multiple studies.

Normal distribution of the parameter estimates from different studies is commonly assumed in the fixed-effect model and random-effects model for traditional meta-analysis. Simulations have shown these methods are relatively robust even under extreme violations of distributional assumptions in estimating heterogeneity [23] and calculating an overall effect size in traditional meta-analysis [24]. However, many meta-analyses include a few studies (such as 5 studies) and such a sample is more inadequate to accurately estimate heterogeneity. In the cases with limited studies, one can still use the weighted LRT method using drug exposure as weight for safety signal exploration. Note that the weight could be study sample size, drug exposure, or could also be defined by the researchers to reflect the importance of the different studies or other study features.

The proposed LRT methods are mainly for postmarket safety evaluation using adverse event data collected from different studies (such as completed clinical trials or observational studies) and for safety signal monitoring using data from ongoing clinical trials. When analyzing observational data from passive surveillance system such as FAERS, we do not have exposure information including total of subjects taking drugs, drug exposure time, and dose. Therefore, we can only evaluate reporting rate, and the denominator for the rate calculation is *n*_*i*._. When analyzing data from clinical trials, we usually have some information about exposure including the number of patients, the dose for each patient, and the exposure time from taking drug to event. Therefore, we can evaluate the risk with denominator *P*_*i*_. When using the proposed method for combining information from multiple studies, one cannot simply combine information from observational data and clinical trial data. In a meta-analysis, we only apply the proposed method to studies with similar features, such as similar denominators, patient populations, study objectives, and so on.

The proposed LRT methods output the *p* values, which incorporate information of relative risk (rr) and exposure from different studies. For each study, an AE signal with higher rr and bigger exposure value may lead to a small *p* value; and an AE with higher rr and small exposure value may not have a small *p* value. The integrated AE signals with small *p* values from all studies are affected by the combined information of the rr estimates and exposure information from all studies. Both relative risk and exposure by study are important information that can be included in the output in addition to the *p* values from the proposed LRT method.

Note that there are missing data issues in data collected from surveillance system (such as delayed reporting, missing reporting, and repeated reporting). There are also missing data issues in clinical trials. For example, patients may drop off before the completion of the one study with less adverse events reported and those patients may have worse disease status compared with patients completing the study. In these situations, we will only observe the available adverse events before drop-off and miss many adverse events after drop-off. We may miss some reports for the patients' missing visits in clinical trials. Some studies may have less missing values and some may have more. In data mining for safety signals, safety investigators usually ignore those missing events in the analysis for the signals. Signals detected without considering missing data in single study or multiple studies may introduce bias. This may be a topic for future research.

## Figures and Tables

**Table 1:** Statistics in different methods (*j* is fixed and drop suffix *j* in the following formulation). Either logLR or LR can be used. In addition to the most significant signal, secondary signals can also be identified.

Method	logLR or weighted logLR	Test statistic (STAT)	Most significant signal detected
Pooled	logLR_*i*_	MLLR=max_*i*_(log(LR_*i*_))	a row
MMLR	logLR_*is*_	MMLLR=max_*s*_ max_*i*_(log(LR_*is*_))	a row-study combination
wLRn	wLR_*i*_=(∑_*s*=1_^*S*_*i*_^*P*_*is*_ logLR_*is*_)/(∑_*s*=1_^*S*_*i*_^*P*_*is*_)	MwLR=max_*i*_(wLR_*i*_)	a row

**Table 2:** Summary of basic information of the PPIs study by trials (rr is for PLandPPI vs. PL).

Study	PL			PL and PPI			rr (95% CI)
	*N* (subject)	Event	Exposure (person-day)	*N* (subject)	Event	Exposure (person-day)	
1st occurrence of AEOST analyses							
*s* = 1	309	14	168947	25	1	10225	1.18 (0.16, 8.97)
*s* = 2	1647	77	1534190	245	20	181753	2.19 (1.34, 3.58)
*s* = 3	210	11	133813	20	2	9822	2.48 (0.55, 11.17)
*s* = 4	1058	167	470833	165	30	56845	1.49 (1.01, 2.19)
*s* = 5	950	144	713856	32	8	19575	2.03 (0.99, 4.13)
*s* = 6	150	17	87481	9	3	2884	5.35 (1.57, 18.26)
All s	4324	430	3109120	496	64	281104	1.65 (1.27, 2.14)
Repeated occurrences of AEOST analyses							
*s* = 1	309	30	173796	25	4	10383	2.23 (0.78, 6.33)
*s* = 2	1647	179	1575058	245	67	196698	3.00 (2.26, 3.97)
*s* = 3	210	13	136128	20	3	10381	3.03 (0.86, 10.62)
*s* = 4	1058	228	513109	165	48	64764	1.67 (1.22, 2.28)
*s* = 5	950	169	789274	32	14	24188	2.70 (1.57, 4.66)
*s* = 6	150	21	95959	9	5	4266	5.36 (2.02, 14.20)
All s	4324	640	3283324	496	141	310680	2.33 (1.94, 2.79)

**Table 3:** Results of PPIs data (for PLandPPI vs. PL). obsstat is the statistics shown in Table 1 for different methods, obtained from the observed data.

	1st occurrence of AEOST			Repeated occurrences of AEOST		
	obsstat	*p* value	95% threshold	obsstat	*p* value	95% threshold
Individual study analysis using regular LRT						
*s* = 1	0.12	0.99	1.79	0.93	0.438	1.96
*s* = 2	4.16	0.004	2.10	24.28	0	2.00
*s* = 3	0.56	0.61	1.73	1.18	0.243	1.18
*s* = 4	1.83	0.07	2.04	4.63	0.006	2.03
*s* = 5	1.54	0.137	1.69	4.88	0.006	2.27
*s* = 6	2.43	0.031	0.97	3.97	0.009	1.17
Simple pooled analysis using regular LRT						
	6.12	0.003	1.78	34.27	0.0	1.83
MMLR						
	4.16	0.006 (from *s* = 2)	2.43	24.28	0 (from *s* = 2)	2.47
wLRn						
	2.81	0.019	1.92	14.02	0	2.12

**Table 4:** Analysis of Lipiodol data (individual study analysis). Bold AE terms are in the PES group.

Studies	# signals	AE terms	obsstat	*p* value	95% threshold
*s* = 1	0	None			3.33
*s* = 2	4	**ABDOMINAL PAIN**	171.8	0	3.93
		**FEVER**	157.5	0	
		ANOREXIA/LOSS OF APPETITE	130.4	0	
		**VOMITTING**	11.6	0	
*s* = 3	3	**FEVER**	192.9	0	4.50
		**ABDOMINAL PAIN**	56.7	0	
		**NAUSEA**	8.5	0	
*s* = 4	2	**FEVER**	7.8	0	4.77
		**ABDOMINAL PAIN**	4.8	0.003	
*s* = 5	3	**FEVER**	29.4	0	4.72
		**ABDOMINAL PAIN**	6.7	0	
		**NAUSEA**	4.73	0.007	
*s* = 6	1	**POST EMBOLIZATION SYNDROME**	29.6	0	3.73
*s* = 7	1	SHOULDER PAIN	32.2	0	4.89
*s* = 8	2	**VOMITTING**	23.6	0	4.89
		**ABDOMINAL PAIN**	7.3	0	
*s* = 9	4	HEMATOLOGICAL or BONE MARROW TOXICITY	34.1	0	4.02
		**FEVER**	9.3	0	
		BILIRUBIN RELATED ABNORMALITIES	4.7	0.015	
		HEPATIC PEDICULITIS	4.7	0.015	
*s* = 10	3	PAIN NOS	26.8	0	3.47
		**FEVER**	24.1	0	
		**VOMITTING**	16.7	0	
*s* = 11	2	**FEVER**	121.8	0	3.73
		PLUERAL EFFUSION	15.5	0	
*s* = 12	1	**FEVER**	4.5	0.008	4.38
*s* = 13	1	RESPPIRATORY DISTURBANCE	3.3	0	3.18

**Table 5:** Analysis of Lipiodol data from multiple studies (integrated results over studies).

Analysis	# signals	AE term (obsstat)	95% threshold
Simple pooled analysis	4	**FEVER** (473.6)	3.93
		**ABDOMINAL PAIN** (195.2)	
		ANOREXIA/LOSS OF APPETITE (61.0)	
		**VOMITTING** (10.7)	
wLRn	4	**FEVER** (89.7), **ABDOMINAL PAIN** (66.8)	1.56
		ANOREXIA/LOSS OF APPETITE (44.0)	
		**VOMITTING** (5.9)	
MMLR	21^*a*^	**FEVER** (192.9), **ABDOMINAL PAIN** (171.8)	6.40
		ANOREXIA/LOSS OF APPETITE (130.4)	
		HEMATOLOGICAL/BONE MARROW TOXICITY (34.1)	
		SHOULDER PAIN (32.2)	
		**POST EMBOLIZATION SYNDROME** (29.6)	
		PAIN NOS(30.0), VOMITTING (26.8)	
		PLUERAL EFFUSION (18.2), **NAUSEA** (8.5)	

*Note*. 21 AE-study combination signals detected by MMLR method, *p* value is 0 for the most significant one (for AE FEVER and 3rd study (*s* = 3)). 10 AE terms were reported in those signals ignoring the study information and are shown in the column for AE term with maximum observed logLR over the studies.

**Table 6:** Type-I error (or FDR) for cases with data generated under null hypothesis (all relative risks to be 1) and power (%) for cases with data generated under alternative hypothesis (varying relative risks).

Cases	Description	LRT methods		
		Pooled	wLRn	MMLR
Type-I error				
sim0allocc	With *p*_*is*_ and *n*_.*s*_ from real data	0.055	0.047	0.050
	Allocc case in Table 2, rr1			
sim01occ	With *p*_*is*_ and *n*_.*s*_ from real data	0.073	0.047	0.049
	1st occurrence case in Table 2, rr1			
sim0LIP	With *p*_*is*_ and *n*_.*s*_ from real Lipiodol	0.046	0.045	0.038
	Data, 13 studies, 27 rows, rr1			
Power				
Simaallocc	rr2	86.7	67.2	48.9
Simaallocc	rr3	99.3	98.4	92.8
Simaallocc	rr4	100	100	99.9
sima1occ	rr2	74.3	37.2	30.1
sima1occ	rr3	97.5	77.0	66.5
sima1occ	rr4	100	98.2	96.9
simaLIP	rr2	38.3	23.4	11.1
simaLIP	rr3	95.0	86.3	63.0
simaLIP	rr4	100	100	99.3
Simaallocc	rr21	32.9	100	99.9
sima1occ	rr21	16.7	11.5	8.0

*Note.* rr1, rr2, rr3, and rr4 are for cases having the 1st row with relative risk (*η*_1_) to be 1, 1.5, 2, and 3, respectively (all other rows have relative risk as 1). In rr1, rr2, rr3, and rr4 cases, *η*_1_ is the same for all 6 studies. In rr21 case, *η*_1_=1.5, 1.2, 3,1,1.3, 2, for 1st row in studies 1 to 6, respectively. For case sim01occ, *n*_1*s*_(=1,20,2,30,8,3) are the counts of the 1st AEOSET events for PLandPPI group from studies 1 to 6, respectively. *P*_1*s*_(=10225,181753,9822,56845,19575,2884) are the exposure for PLandPPI group from studies 1 to 6, respectively. *n*_2*s*_(=14,77,11,167,144,17) are the counts of 1st AEOSET events for PL group from studies 1 to 6, respectively. *P*_2*s*_(=168947,1534190,133813,470833,713856,87481) are the exposure for PL group from studies 1 to 6, respectively. The *n*_*is*_ and *P*_*is*_, *i*=1,2 for case sim0allocc can also be found in Table 2 (for repeated occurrences of AEOST). For case sim0LIP, *n*_*is*_ and *P*_*is*_(*i*=1, ⋯, 27 and *s*=1, ⋯, 13) cannot be listed here due to space limitation. The values of *n*_*is*_ range from 0 to 128, and the values of *P*_*is*_=*P*_*s*_ range from 11 to 257.

## Data Availability

The hypothetical data for PPI analysis and the Lipiodol data used to support the findings of this study are available from the corresponding author upon request.
